# Prediction of G Protein-Coupled Receptors with SVM-Prot Features and Random Forest

**DOI:** 10.1155/2016/8309253

**Published:** 2016-07-27

**Authors:** Zhijun Liao, Ying Ju, Quan Zou

**Affiliations:** ^1^School of Basic Medical Sciences, Fujian Medical University, Fuzhou, Fujian 350108, China; ^2^School of Computer Science and Technology, Tianjin University, Tianjin 300350, China; ^3^School of Information Science and Technology, Xiamen University, Xiamen, Fujian 361005, China; ^4^State Key Laboratory of Medicinal Chemical Biology, Nankai University, Tianjin 300071, China

## Abstract

G protein-coupled receptors (GPCRs) are the largest receptor superfamily. In this paper, we try to employ physical-chemical properties, which come from SVM-Prot, to represent GPCR. Random Forest was utilized as classifier for distinguishing them from other protein sequences. MEME suite was used to detect the most significant 10 conserved motifs of human GPCRs. In the testing datasets, the average accuracy was 91.61%, and the average AUC was 0.9282. MEME discovery analysis showed that many motifs aggregated in the seven hydrophobic helices transmembrane regions adapt to the characteristic of GPCRs. All of the above indicate that our machine-learning method can successfully distinguish GPCRs from non-GPCRs.

## 1. Introduction

The G protein-coupled receptors (GPCRs) are only discovered in eukaryotes, which constitute a vast protein family and perform their various functions always through coupling with G proteins in the cell. GPCRs have many aliases such as heptahelical receptors, serpentine receptor, G protein-linked receptors (GPLR), and seven-transmembrane (7TM) domain receptors; all the GPCRs contain a single polypeptide chain that pass through the cell membrane seven times [1]. There are roughly 1000 GPCRs in human genome (accounting for about 2% coding genes); thus, they form the largest receptor superfamily [2]; they are also involved in various diseases and constituted approximately 40% of drug targets. Because Robert J. Lefkowitz and Brian K. Kobilka revealed the biochemical mechanism of GPCRs for signaling pathways, they were awarded with 2012 Nobel Prize in chemistry [3].

Many different approaches have been utilized for GPCRs classification, such as protein motif-based systems, machine-learning methods [4], and other techniques. Based on the original sequence similarity and phylogenetic studies, GPCRs superfamily can be divided into five, six, or seven classes at different periods [5, 6]. According to GPCRdb (http://gpcrdb.org/) database developed by Kolakowski and updated by Horn et al. [7], which contains data, diagrams, and web tools involving collection of both GPCRs crystal structures and receptor mutants, GPCRs are classified into six main families: class A (Rhodopsin), class B1 (Secretin), class B2 (Adhesion), class C (Glutamate), class F (Frizzled), and other GPCRs. The former five classes are consistent with the Glutamate, Rhodopsin, Adhesion, Frizzled, and Secretin (GRAFS in short) classification system [8, 9].  Table 1 shows the protein number and composition for every class.

Class A rhodopsin-like receptors constitute the largest (more than 80%) of the human GPCR subtypes. They mediate numerous effects of endogenous peptides including neurotransmitters, hormones, and paracrine signals. For example, biogenic amines [10] such as norepinephrine, dopamine, and serotonin commonly play their role of drugs for pathological diseases through binding to GPCRs. Although the N-terminal extracellular domain is very short, class A receptors can form dimers, in homo/heterodimerization [11]. This class also includes approximately 60 orphan receptors which have no defined ligands or functions at all [12, 13].

Class B1 secretin-like receptors belong to one of hormone and neuropeptide receptor families; they consist of a large and versatile N-terminal extracellular domain (ECD) which functions as an affinity trap to hormone [14]. Moreover, they are of ancient origin and can bind with various peptides such as secretin, corticotrophin releasing factor, glucagon, parathyroid hormone, calcitonin, growth hormone releasing hormone, and calcitonin gene-related peptide [15].

Class B2 adhesion-like receptors are also known as the adhesion G protein-coupled receptors (ADGRs) with ancient origin; they make the function in various tissues include synapses of the brain [16]. Most ADGRs contain various domains in the N-terminus provided for binding site of other cells [17]; these domains have over sixteen types, including cadherin-like repeats, thrombospondin-like repeats, and calnexin domain. ADGRs have the characteristic of N-terminal adhesive domains [18]. For example, ADGR subfamily G4 (ADGRG4) has the sequence characteristics of a unique highly conserved motif and some functionally important motifs similar to class A, class B1, and combined elements [19].

Class C GPCRs mainly comprise metabotropic glutamate receptors (mGluRs), one type of L-glutamate binding receptors; another type is ionotropic glutamate receptors (iGluRs) which belong to a ligand-gated ion channels not the GPCR family. Class C GPCRs contain a large N-terminal domain for ligand-binding. There exist 8 isoforms of mGluRs to form signaling molecules via second messenger systems [20], which transfer extracellular signal through the mechanism of receptor dimer packing and allosteric regulation [21]. The activation of mGluRs is an indirect metabotropic process by the aid of binding to glutamate, a major excitatory neurotransmitter in the brain. The extracellular glutamate concentration (at micromolar range) is lower than the intracellular (at millimolar range) in neuron [22]. Human mGluRs are found in pre- and postsynaptic neurons, including the hippocampus, cerebellum, and other brain regions' synapses, and in peripheral tissues. mGluRs play an important role in regulating neuronal excitability and synaptic plasticity and in serving as mental disorders drug targets [23].

Class F frizzled/smoothened receptors are involved in Wnt binding whereas the smoothened receptor (belongs to GPCRs) reconciles hedgehog signaling via the required region cysteine-rich domain (CRD) in the N-terminus [24], because smoothened protein sequence is homologous to frizzled. The two proteins have the same 7TM structure and evolutionary relationship [25]. But the secreted frizzled-related proteins can exert its function by promoting or blocking Wnt3*α*/*β*-catenin signaling in different concentration of secreted frizzled-related protein 1 and cellular context [26].

Other GPCRs include some orphan receptors except for the above classes; the characteristics of these receptors are that they have a similar structure to other identified receptors but lack endogenous ligand. They have altogether 37 proteins and 6 in human. Among them, Gpr175 (also called Tpra1) and GPR157 are well studied. Gpr175 is an orphan GPCR with positive regulation of the Hedgehog signaling pathway [27]; GPR157 couples with Gq protein and then activate IP_3_-mediated Ca^2+^ cascade, which is also a signaling molecule involved in positive regulation of neuronal differentiation of radial glial progenitors through the GPR157-Gq-IP_3_ cascade pathway [28].

Generally, GPCRs interact with a varieties of ligands which can be classified as agonists, antagonists, or inverse agonists, three classes based on the receptor effect [29, 30]; these include different forms of “information,” such as photons, taste, odorants [31], ions, pheromones, eicosanoids, nucleotides, nucleosides [9], neurotransmitters, amino acids [32], peptides, proteins, and hormones [33]. These ligands vary in size containing small molecules and large proteins.

GPCRs are transmembrane receptors that transduce extracellular stimuli into intracellular signals through activating intracellular heterotrimeric G protein complex, which comprise 15 G*α* subunits, 5 G*β* subunits, and 12 G*γ* subunits. Based on the sequence similarity and functional characteristics of G*α* subunits, G proteins are divided into four major classes: G*α*s, G*α*i/o, G*α*q/11, and G*α*12/13 [34]. G*α* activation or deactivation cycle controls the signal transduction, when cell is at resting mode, GDP binds to G*α* forming G*α*-GDP and then joins G*βγ* generating G*αβγ* complex, and G*α* is inactive at this stage; when stimulate signal is introduced from GPCR, G*α* raises a conformational change, GTP binds to G*α* forming G*α*-GTP and destabilizing the G*αβγ* complex, G*βγ* are disassociated and bound by G*βγ* interacting proteins, and G*α* is active at this stage. When G*α* fulfilled signal transduction to the downstream pathway, G*α* hydrolyzes GTP to GDP through its intrinsic GTPase activity to form G*α*-GDP and returns to the resting mode; this process constitutes a G protein cycle [35]. Activated G*α*s catalyzes ATP to cAMP by adenylyl cyclase (AC) and results in the activation of protein kinase A (PKA) and phosphorylation of downstream effector. On the contrary, G*α*i plays inhibition role of AC and suppresses cAMP production. G*α*q/11 activates phospholipase C*β* (PLC*β*) and produces inositol-1,4,5-trisphosphate (IP_3_) and diacylglycerol (DAG) which can form PLC*β*-IP_3_-DAG signaling pathway. G*α*12/13 activates Rho GTPase families through RhoGEF to regulate cytoskeleton remodeling; these G protein families take the major effect in signal transduction [3]. Therefore, GPCR-G*α*-AC-PKA and GPCR-G*α*-PLC-IP_3_ constitute two main signal transduction cascades within the cell.

In this paper, we performed an* in silico* analysis on the GPCRs amino acids information and other polypeptide physicochemical features and constructed 188D feature vectors (Table 2) of the proteins into an ensemble classifier [36–41]. The first 20D of 188D represents the 20 kinds of natural amino acids composition; the other 168D includes eight physical-chemical properties each deriving from the so-call CTD mode [42], where C stands for amino acid contents for each type of hydrophobic amino acids, T stands for the frequency of bivalent peptide, and D stands for amino acid distribution from five positions of a sequence. These 188D feature vectors have been integrated into software BinMemPredict which performed well in membrane protein prediction [42]. Moreover, we also performed motif analysis by MEME Suite (http://meme-suite.org/) because a motif may directly accord with the active site of an enzyme or a domain of the protein. MEME have been not only used to predict conserved motif regions but also employed for primers design with low quality sequence similarity patterns in multiple global alignments [43].

## 2. Materials and Methods

### 2.1. Data Retrieval and Pretreatment

GPCR sequences with fasta format were retrieved from the UniProt database (http://www.uniprot.org/); we obtained initial 5027 sequences altogether. To improve analysis performance, the raw dataset was preprocessed by the protein-clustering program CD-HIT (http://cd-hit.org/) for reducing the sequence homology bias of prediction; the sequence identity threshold was set at 0.80 and other parameters as default; thus, the highly homology sequences were removed, and finally 2495 GPCR protein sequences were gained as positive dataset, and the negative examples were from all the protein sequences but removing the positive ones, and 10386 entries (non-GPCRs) were acquired as negative dataset.

### 2.2. Extracting the Discriminative Feature Vector for Classifying and Testing by Random Forest Classifier

Protein features were extracted from the primary sequences according to their compositions of 20 kinds of amino acids and their eight types of physical-chemical properties; based on these characteristics, Cai et al. [44] and Zou et al. [42] had raised 188D feature vectors of SVM-Prot. The workflow was as follows:

(1) All distinct positive protein samples were employed to extract their corresponding protein families for Pfam number from the “Family and Domains” section of uniprot website and excluded the same and redundant Pfam number; the unique Pfam number set for positive dataset (in fasta format) was acquired.

(2) All the protein sequences were integrated into a Pfam number file; the same Pfam sequences were combined to the same file named with Pfam number; then, the positive Pfam number files were removed; the rest of Pfam number files were extracted only in the longest sequence for each Pfam as the negative dataset (in fasta format).

(3) Because the protein sequences possessed different length, each sequence needed to transform into fixed-size vectors for classification, both the positive and negative datasets were input to the 188D SVM-Prot programme for their feature vectors, the positive samples were given the label “1” at the end of vectors, the negative samples were given the label “−1” at the end of vectors, and the positive and negative files combined into a file with the filename format ended in  .arff.

(4) The above file on positive and negative vector datasets was randomly divided into five parts, respectively, among which, every four parts were served as training examples and the remaining one part as test ones, every part contained both positive and negative samples (Table 3), and fivefold cross-validation was used.

(5) The training and test datasets were successively imported into weka data mining package (http://www.cs.waikato.ac.nz/ml/weka/), a machine-learning workbench. In weka, the training datasets were filtered with the synthetic minority oversampling technique (SMOTE) [45, 46] and changed the positive samples from 100 percent into 300 percent to overcome the highly imbalanced property of positive and negative cases; after preprocessing with SMOTE technique the two-group data kept an amount equilibrium, and the vector data were classified automatically via visualization analysis [47]. Based on the optimal features with some preliminary trials, we finally chose a Random Forest (RF) [48] module and “use training set” item on test options as classifier for training dataset, while for test dataset we chose “supplied test set” item on test options to predict the samples as GPCRs or non-GPCRs: that is, the prediction module using the results of the just training set to distinguish the two classes.

To measure the performance quality of the statistical classification more intuitively in the field of machine learning, we adopted 5-fold cross-validation for test dataset and calculated four common parameters [49, 50]: sensitivity (Sn), specificity (Sp), accuracy (Acc), and Matthew's correlation coefficient (MCC) to adopt for evaluating the SVM-Prot features and classifier, which are formulated as Table 4.

### 2.3. Conserved Motif Analyses of Human GPCR Proteins

Online MEME Suite 4.11.0 (http://meme-suite.org/) was used to analyze conserved motif analyses. MEME was a powerful, comprehensive web-based tool for mining sequence motifs in proteins, DNA, and RNA [51]. Currently, the MEME Suite has added 6 new tools since the* Nucleic Acids Research Web Server Issue* in 2009, and the web-based version tools reached 13. The maximum motif width, the minimal motif width, and the maximum number of motifs were set to 50, 6, and 10, respectively.

## 3. Results

### 3.1. Reclassification of Positive and Negative Proteins on Five Test Datasets

We obtained the 188D feature vectors containing positive and negative samples and divided them into training and test datasets as input to the Weka explorer, respectively, the results showed exactly classifying for all the five training datasets; therefore, the trained classifier could be utilized to verify the predication effect, and the test dataset was used to predict its class label directly. The correctly classified rates for five testing datasets were 90.64%, 90.37%, 88.04%, 93.28%, and 95.73%, respectively (mean ± SD: 91.61% ± 2.96%); the other indices were shown in Table 5.

### 3.2. Conserved Motifs Analysis for Human GPCRs

**Figure 1:**
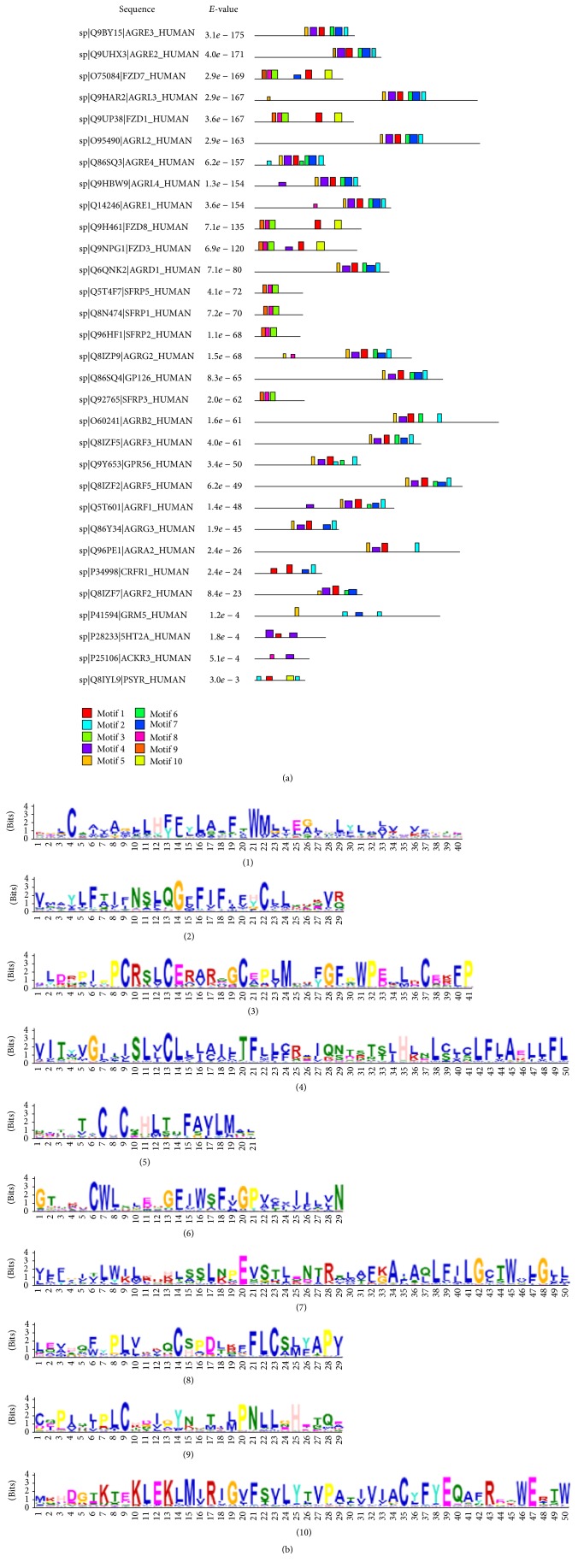
The discovered motifs of human GPCRs from the MEME system (for details see Table 6). (a) MEME run showing combined block diagram for top ten motifs distribution with corresponding sequence ID and* E*-value (*E*-value threshold: 0.01, showing 31 GPCR sequences). (b) The ten motif logos found by MEME.

For the purpose of disclosing the evolutionary relationship of the conserved motifs of GPCRs, we randomly selected six classes of human GPCRs and gained 66 protein sequences which were analyzed by MEME software. The multiple local alignments were performed by MEME to generate the most significant 10 conserved motifs for the sequences (Figure 1 and Table 6).

## 4. Discussion

In this study we show that the novel SVM-Prot features based binary classifier can well discriminate GPCRs from non-GPCRs; we obtain exact classification model from the five training datasets and the AUC equals 1, and on the five testing datasets we get the average correctly classified rates of 91.61% and the average AUC of 0.9282; these indicate that predicted GPCRs and true GPCRs have a good overall consistency. AUC is a plot with *x*-axis representing false positives (equal to 1 − specificity) and *y*-axis representing true positives (equal to sensitivity), which is based on different cutoff values of a score from a binary classifier [52, 53]. AUC of 1 represents a perfect model; the more AUC is close to 1, the better prediction model we can develop, but if the value is reduced to 0.5, the model becomes no predictive ability at all. On our binary classification model we acquired high specificity and accuracy for testing datasets, but the values of sensitivity and Matthew's correlation coefficient were relatively low at about 0.7; this might be due to the problem of imbalance dataset where the size of positive was less than negative with the proportion of about 1 : 4; thus the false negative rate was relatively higher. This defect may also come from the intrinsic restriction of supervised learning algorithm, because the classification model built from training dataset can only have a good predictive effect on the test dataset having the same probability distribution as the training dataset [54].

The top ten human GPCR motifs show the feature of some motifs aggregation that appeared from the block diagram; this reflected in the structure characteristic of 7TM helices regions of GPCRs. Motifs 1,4,6,7, and 10 belonged to these 7TM domains; among them, the former 4 motifs displayed containing the region highly homologous to the class B1 secretin family, and motif 10 was a Fz domain in the membrane spanning region which is located near to the intracellular C-terminal region of GPCRs, which contained an alpha-helical Cys-rich domain (CRD) of Frizzled that was essential for Wnt binding [55, 56]. Motifs 3, 8, and 9 were CRD Frizzled-1 like domains involved in Wnt signal as well [57]. Motif 5 was latrophilin/CL-1-like G protein–coupled receptor proteolysis site motif (GPS) which was first identified in a neuronal Ca^2+^-independent receptor of alpha-latrotoxin (CIRL)/latrophilin, an orphan GPCR [58]. GPS was a part of GPCR autoproteolysis-inducing (GAIN) domain which held a formative feature of adhesion GPCRs, and GPS cleavage process played an important role in renal organ physiology [59]. Take the first sequence Q9BY15, for instance, there listed 3 kinds of conserved domains start from the N-terminus: calcium-binding EGF domain (not shown), GPS domain, and 7TM domain of secretin family. The latter two domains appeared with concentration on the block diagram.

Support Vector Machine (SVM) is a supervised machine-learning algorithm on the basis of statistical learning theory [53, 60–65]. Due to the robustness, rapidness, and repeatability, machine-learning method is regarded as one of the best ways to efficiently classify numerous protein molecules. In two-class problems, our SVM classifier mapped the input 188D feature vectors into a higher dimensional feature space and then founded the optimal separation hyperplane [66] for GPCRs and non-GPCRs, while avoiding overfitting and underfitting problems. This approach belongs to linear classification model [67].

All the GPCR superfamily contains seven highly conserved 7TM regions with the feature of hydrophobicity; these 7TM can be identified by Hidden Markov Models (HMMs) and machine-learning methods [68]. The GPCRs structure researchers revealed that the classical sequence contained the following: the seven-transmembrane segments [TM1–7], three extracellular loops [EL1–3], three intracellular loops [IL1–3], and the protein termini. Therefore, GPCR can be sequentially distributed into the following regions: N-terminus-TM1-IL1-TM2-EL1-TM3-IL2-TM4-EL2-TM5-IL3-TM6-EL3-TM7-C terminus. In summary, we have successfully developed a SVM-Prot features based Random Forest for identifying GPCRs from non-GPCRs based on the protein sequence information and their physicochemical properties. Nevertheless, this prediction model needs to be further explored so as to discriminate the subfamily and sub-subfamily of GPCRs.

## Figures and Tables

**Table 1:** The number of proteins and composition for every class of GPCRs (from GPCRdb).

GPCRdb family	Number of proteins (human)	Composition
Class A (rhodopsin)	16526 (311)	Aminergic receptors, peptide receptors, protein receptors, lipid receptors, melatonin receptors, nucleotide receptors, steroid receptors, alicarboxylic receptors, sensory receptors, orphan receptors, and others
Class B1 (secretin)	748 (15)	Peptide receptors
Class B2 (adhesion)	381 (33)	Orphan receptors
Class C (glutamate)	1038 (22)	Ion receptors, amino acid receptors, sensory receptors, and orphan receptors
Class F (frizzled)	48 (11)	Peptide receptors
Other GPCRs	37 (6)	Orphan receptors

**Table 2:** The composition of 188D features of a protein.

Physicochemical property	Dimensions
Amino acid composition	20
Hydrophobicity	21
Normalized Van der Waals volume	21
Polarity	21
Polarizability	21
Charge	21
Surface tension	21
Secondary structure	21
Solvent accessibility	21

Total	188

**Table 3:** The distribution of positive and negative sample numbers for training and test dataset.

Performance	Part	Number of GPCRs	Number of non-GPCRs	Total number
1st	Training	1996	8309	10305
1st	Test	499	2077	2576
2nd	Training	1996	8309	10305
2nd	Test	499	2077	2576
3rd	Training	1996	8309	10305
3rd	Test	499	2077	2576
4th	Training	1996	8309	10305
4th	Test	499	2077	2576
5th	Training	1996	8308	10304
5th	Test	499	2078	2577

**Table 4:** Performance measures for random forest from SVM-Prot feature.

Measure	Formula	Meaning
Sensitivity	$\text{Sn}=\frac{\text{TP}}{\text{TP}+\text{FN}}$	Measure to avoid type II error
Specificity	$\text{Sp}=\frac{\text{TN}}{\text{TN}+\text{FP}}$	Measure to avoid type I error
Accuracy	$\text{Acc}=\frac{\text{TP}+\text{TN}}{\text{TP}+\text{FP}+\text{TN}+\text{FN}}$	Measure of correctness
Matthew's correlation coefficient	$\text{MCC}=\frac{\text{TP}*\text{TN}-\text{FP}*\text{FN}}{\sqrt{\left(\text{TP}+\text{FN}\right)\left(\text{TP}+\text{FP}\right)\left(\text{TN}+\text{FP}\right)(\text{TN}+\text{FN})}}$	Correlation coefficient

TP (true positive) stands for the number of true GPCRs that are predicted correctly, TN (true negative) stands for the number of true non-GPCRs that are predicted correctly, FP (false positive) is the number of true non-GPCRs that are incorrectly predicted to be GPCRs, and FN (false negative) is the number of true GPCRs that are incorrectly predicted to be non-GPCRs.

**Table 5:** Performance qualities measure for test dataset by using the models from the corresponding training dataset.

Test dataset	Sn	Sp	Acc	MCC	AUC^*∗*^
1st	0.5952	0.9812	0.7882	0.6248	0.930
2nd	0.5832	0.9807	0.7820	0.6146	0.909
3rd	0.6013	0.9620	0.7817	0.5763	0.879
4th	0.7675	0.9726	0.8700	0.7562	0.943
5th	0.9238	0.9654	0.9446	0.8900	0.980
Mean ± SD	0.6942 ± 0.1491	0.9724 ± 0.0087	0.8333 ± 0.0726	0.6924 ± 0.1296	0.928 ± 0.038

^*∗*^AUC, also called receiver operating characteristic (ROC) area, means the area under the receiver operating characteristic curve which is a measure of the accuracy of a classification model.

**Table 6:** Human top 10 conserved motifs of GPCR sequences found by the MEME system.

Motif	Width	*E*-value	Best possible match
1	40	4.3*e* − 239	KMACTIMAMFLHYFYLAAFFWMLIEGLHLYLMAVMVWHHE
2	29	1.5*e* − 168	VMHYLFTIFNSFQGFFIFIFHCLLNRQVR
3	41	4.4*e* − 105	CLDRPIPPCRSLCERARQGCEPLMNKFGFPWPEMMKCDKFP
4	50	5.3*e* − 098	VITWVGIIISLVCLLICIFTFLFCRAIQNTRTSIHKNLCICLFLAHLLFL
5	21	3.8*e* − 088	NKTHTTCRCNHLTNFAVLMAH
6	29	1.0*e* − 076	GTDKRCWLHLDKGFIWSFIGPVCVIILVN
7	50	3.9*e* − 063	IFFIITLWIMKRHLSSLNPEVSTLQNTRMWAFKAFAQLFILGCTWCFGIL
8	29	1.8*e* − 054	LQVHQWYPLVKKQCHPDLKFFLCSMYAPV
9	29	1.6*e* − 052	CQPIDIPLCHDIGYNQMIMPNLLNHETQE
10	50	2.0*e* − 052	MKHDGTKTEKLEKLMIRIGVFSVLYTVPATIVIACYFYEQAFRDHWERTW

## References

[1] Trzaskowski B., Latek D., Yuan S., Ghoshdastider U., Debinski A., Filipek S. (2012). Action of molecular switches in GPCRs—theoretical and experimental studies. *Current Medicinal Chemistry*.

[2] Shore D. M., Reggio P. H. (2015). The therapeutic potential of orphan GPCRs, GPR35 and GPR55. *Frontiers in Pharmacology*.

[3] Lin H.-H. (2013). G-protein-coupled receptors and their (Bio) Chemical significance win 2012 nobel prize in chemistry. *Biomedical Journal*.

[4] Zou Q. (2016). Machine learning techniques for protein structure, genomics function analysis and disease prediction. *Current Proteomics*.

[5] Que Y., Xu L., Wu Q. (2014). Genome sequencing of Sporisorium scitamineum provides insights into the pathogenic mechanisms of sugarcane smut. *BMC Genomics*.

[6] Rosenbaum D. M., Rasmussen S. G. F., Kobilka B. K. (2009). The structure and function of G-protein-coupled receptors. *Nature*.

[7] Horn F., Bettler E., Oliveira L., Campagne F., Cohen F. E., Vriend G. (2003). GPCRDB information system for G protein-coupled receptors. *Nucleic Acids Research*.

[8] Krishnan A., Almén M. S., Fredriksson R., Schiöth H. B. (2012). The origin of GPCRs: identification of mammalian like rhodopsin, adhesion, glutamate and frizzled GPCRs in fungi. *PLoS ONE*.

[9] Kochman K. (2014). Superfamily of G-protein coupled receptors (GPCRs)—extraordinary and outstanding success of evolution. *Postepy Higieny i Medycyny Doswiadczalnej*.

[10] Balfanz S., Jordan N., Langenstück T., Breuer J., Bergmeier V., Baumann A. (2014). Molecular, pharmacological, and signaling properties of octopamine receptors from honeybee (*Apis mellifera*) brain. *Journal of Neurochemistry*.

[11] Franco R., Martínez-Pinilla E., Lanciego J. L., Navarro G. (2016). Basic pharmacological and structural evidence for Class A G-protein-coupled receptor heteromerization. *Frontiers in Pharmacology*.

[12] Shepard B. D., Natarajan N., Protzko R. J., Acres O. W., Pluznick J. L. (2013). A cleavable N-terminal signal peptide promotes widespread olfactory receptor surface expression in HEK293T cells. *PLoS ONE*.

[13] Sreedharan S., Almén M. S., Carlini V. P. (2011). The G protein coupled receptor Gpr153 shares common evolutionary origin with Gpr162 and is highly expressed in central regions including the thalamus, cerebellum and the arcuate nucleus. *FEBS Journal*.

[14] Zhao L.-H., Yin Y., Yang D. (2016). Differential requirement of the extracellular domain in activation of class B G protein-coupled receptors. *The Journal of Biological Chemistry*.

[15] Cardoso J. C. R., Pinto V. C., Vieira F. A., Clark M. S., Power D. M. (2006). Evolution of secretin family GPCR members in the metazoa. *BMC Evolutionary Biology*.

[16] Duman J. G., Tu Y.-K., Tolias K. F. (2016). Emerging roles of BAI adhesion-GPCRs in synapse development and plasticity. *Neural Plasticity*.

[17] Hamann J., Aust G., Araç D. (2015). International union of basic and clinical pharmacology. XCIV. Adhesion G protein-coupled receptors. *Pharmacological Reviews*.

[18] Langenhan T., Aust G., Hamann J. (2013). Sticky signaling—adhesion class g protein-coupled receptors take the stage. *Science Signaling*.

[19] Peeters M. C., Mos I., Lenselink E. B., Lucchesi M., IJzerman A. P., Schwartz T. W. (2016). Getting from A to B-exploring the activation motifs of the class B adhesion G protein-coupled receptor subfamily G member 4/GPR112. *The FASEB Journal*.

[20] Spooren W., Lesage A., Lavreysen H., Gasparini F., Steckler T. (2010). Metabotropic glutamate receptors: their therapeutic potential in anxiety. *Current Topics in Behavioral Neurosciences*.

[21] Bai Q., Yao X. (2016). Investigation of allosteric modulation mechanism of metabotropic glutamate receptor 1 by molecular dynamics simulations, free energy and weak interaction analysis. *Scientific Reports*.

[22] Lewerenz J., Maher P. (2015). Chronic glutamate toxicity in neurodegenerative diseases-what is the evidence?. *Frontiers in Neuroscience*.

[23] Vafabakhsh R., Levitz J., Isacoff E. Y. (2015). Conformational dynamics of a class C G-protein-coupled receptor. *Nature*.

[24] Nachtergaele S., Whalen D. M., Mydock L. K. (2013). Structure and function of the Smoothened extracellular domain in vertebrate Hedgehog signaling. *eLife*.

[25] Pei J., Grishin N. V. (2012). Cysteine-rich domains related to Frizzled receptors and Hedgehog-interacting proteins. *Protein Science*.

[26] Xavier C. P., Melikova M., Chuman Y., Üren A., Baljinnyam B., Rubin J. S. (2014). Secreted Frizzled-related protein potentiation versus inhibition of Wnt3a/*β*-catenin signaling. *Cellular Signalling*.

[27] Singh J., Wen X., Scales S. J. (2015). The orphan G protein-coupled receptor Gpr175 (Tpra40) enhances Hedgehog signaling by modulating cAMP levels. *The Journal of Biological Chemistry*.

[28] Takeo Y., Kurabayashi N., Nguyen M. D., Sanada K. (2016). The G protein-coupled receptor GPR157 regulates neuronal differentiation of radial glial progenitors through the Gq-IP_3_ pathway. *Scientific Reports*.

[29] Mathew E., Bajaj A., Connelly S. M. (2011). Differential interactions of fluorescent agonists and antagonists with the yeast G protein coupled receptor ste2p. *Journal of Molecular Biology*.

[30] Kuohung W., Burnett M., Mukhtyar D. (2010). A high-throughput small-molecule ligand screen targeted to agonists and antagonists of the g-protein-coupled receptor GPR54. *Journal of Biomolecular Screening*.

[31] Gonzalez-Kristeller D. C., do Nascimento J. B. P., Galante P. A. F., Malnic B. (2015). Identification of agonists for a group of human odorant receptors. *Frontiers in Pharmacology*.

[32] Thurmond R. L. (2015). The histamine H4 receptor: from orphan to the clinic. *Frontiers in Pharmacology*.

[33] Lv X., Liu J., Shi Q. (2016). In vitro expression and analysis of the 826 human G protein-coupled receptors. *Protein Cell*.

[34] Hildebrandt J. D. (1997). Role of subunit diversity in signaling by heterotrimeric G proteins. *Biochemical Pharmacology*.

[35] Sato M. (2013). Roles of accessory proteins for heterotrimeric G-protein in the development of cardiovascular diseases. *Circulation Journal*.

[36] Yu Z., Li L., Liu J., Han G. (2015). Hybrid adaptive classifier ensemble. *IEEE Transactions on Cybernetics*.

[37] Lin C., Zou Y., Qin J. (2013). Hierarchical classification of protein folds using a novel ensemble classifier. *PLoS ONE*.

[38] Zou Q., Guo J., Ju Y., Wu M., Zeng X., Hong Z. (2015). Improving tRNAscan-SE annotation results via ensemble classifiers. *Molecular Informatics*.

[39] Yu Z., Chen H., Liu J. (2016). Hybrid *κ*—nearest neighbor classifier. *IEEE Transactions on Cybernetics*.

[40] Yu Z., Li L., Liu J., Zhang J., Han G. (2015). Adaptive noise immune cluster ensemble using affinity propagation. *IEEE Transactions on Knowledge and Data Engineering*.

[41] Lin C., Chen W., Qiu C., Wu Y., Krishnan S., Zou Q. (2014). LibD3C: ensemble classifiers with a clustering and dynamic selection strategy. *Neurocomputing*.

[42] Zou Q., Li X., Jiang Y., Zhao Y., Wang G. (2013). BinMemPredict: a web server and software for predicting membrane protein types. *Current Proteomics*.

[43] Sahu M., Sahu J., Sahoo S. (2012). An approach to delineate primers for a group of poorly conserved sequences incorporating the common motif region. *Bioinformation*.

[44] Cai C. Z., Han L. Y., Ji Z. L., Chen X., Chen Y. Z. (2003). SVM-Prot: web-based support vector machine software for functional classification of a protein from its primary sequence. *Nucleic Acids Research*.

[45] Chen K. H., Wang K., Adrian A. M., Teng N. (2016). Diagnosis of brain metastases from lung cancer using a modified electromagnetism like mechanism algorithm. *Journal of Medical Systems*.

[46] Wiharto W., Kusnanto H., Herianto H. (2016). Intelligence system for diagnosis level of coronary heart disease with K-star algorithm. *Healthcare Informatics Research*.

[47] Frank E., Hall M., Trigg L., Holmes G., Witten I. H. (2004). Data mining in bioinformatics using Weka. *Bioinformatics*.

[48] Liu B., Long R., Chou K. (2016). iDHS-EL: identifying DNase I hypersensitive sites by fusing three different modes of pseudo nucleotide composition into an ensemble learning framework. *Bioinformatics*.

[49] Wang R., Xu Y., Liu B. (2016). Recombination spot identification based on gapped k-mers. *Scientific Reports*.

[50] Liu B., Wang S., Dong Q., Li S., Liu X. (2016). Identification of DNA-binding proteins by combining auto-cross covariance transformation and ensemble learning. *IEEE Transactions on NanoBioscience*.

[51] Bailey T. L., Johnson J., Grant C. E., Noble W. S. (2015). The MEME suite. *Nucleic Acids Research*.

[52] Uno K., Yoshizaki K., Iwahashi M. (2015). Pretreatment prediction of individual rheumatoid arthritis patients' response to anti-cytokine therapy using serum cytokine/chemokine/soluble receptor biomarkers. *PLoS ONE*.

[53] Tang H., Chen W., Lin H. (2016). Identification of immunoglobulins using Chou's pseudo amino acid composition with feature selection technique. *Molecular BioSystems*.

[54] Davies M. N., Gloriam D. E., Secker A. (2007). Proteomic applications of automated GPCR classification. *Proteomics*.

[55] Tsukiyama T., Fukui A., Terai S. (2015). Molecular role of RNF43 in canonical and noncanonical Wnt signaling. *Molecular and Cellular Biology*.

[56] Brinkmann E., Mattes B., Kumar R. (2016). Secreted frizzled-related protein 2 (sFRP2) redirects non-canonical Wnt signaling from Fz7 to Ror2 during vertebrate gastrulation. *The Journal of Biological Chemistry*.

[57] Thysen S., Cailotto F., Lories R. (2016). Osteogenesis induced by frizzled-related protein (FRZB) is linked to the netrin-like domain. *Laboratory Investigation*.

[58] Krasnoperov V. G., Bittner M. A., Beavis R. (1997). *α*-Latrotoxin stimulates exocytosis by the interaction with a neuronal G-protein-coupled receptor. *Neuron*.

[59] Trudel M., Yao Q., Qian F. (2016). The role of G-protein-coupled receptor proteolysis site cleavage of polycystin-1 in renal physiology and polycystic kidney disease. *Cells*.

[60] Ma H., Chang W., Cui G. (2012). Ecological footprint model using the support vector machine technique. *PLoS ONE*.

[61] Ding H., Feng P.-M., Chen W., Lin H. (2014). Identification of bacteriophage virion proteins by the ANOVA feature selection and analysis. *Molecular Biosystems*.

[62] Liu B., Zhang D., Xu R. (2014). Combining evolutionary information extracted from frequency profiles with sequence-based kernels for protein remote homology detection. *Bioinformatics*.

[63] Li D., Ju Y., Zou Q. (2016). Protein folds prediction with hierarchical structured SVM. *Current Proteomics*.

[64] Ding H., Guo S.-H., Deng E.-Z. (2013). Prediction of Golgi-resident protein types by using feature selection technique. *Chemometrics and Intelligent Laboratory Systems*.

[65] Yuan L.-F., Ding C., Guo S.-H., Ding H., Chen W., Lin H. (2013). Prediction of the types of ion channel-targeted conotoxins based on radial basis function network. *Toxicology in Vitro*.

[66] Haasdonk B. (2005). Feature space interpretation of SVMs with indefinite kernels. *IEEE Transactions on Pattern Analysis and Machine Intelligence*.

[67] Hinselmann G., Rosenbaum L., Jahn A., Fechner N., Ostermann C., Zell A. (2011). Large-scale learning of structure-activity relationships using a linear support vector machine and problem-specific metrics. *Journal of Chemical Information and Modeling*.

[68] Bouziane H., Messabih B., Chouarfia A. (2011). Profiles and majority voting-based ensemble method for protein secondary structure prediction. *Evolutionary Bioinformatics*.

